# Antimigraine activity of Asarinin by OPRM1 pathway with multifaceted impacts through network analysis

**DOI:** 10.1038/s41598-024-70933-2

**Published:** 2024-08-30

**Authors:** Rapuru Rushendran, Vellapandian Chitra

**Affiliations:** Department of Pharmacology, SRM College of Pharmacy, SRM Institute of Science and Technology, Kattankulathur, Chengalpattu, Tamil Nadu 603 203 India

**Keywords:** Migraine, Calcitonin gene-related peptide, Asarinin, Molecular docking, Molecular dynamics, Network pharmacology, Drug discovery, Neuroscience, Neurology

## Abstract

Migraine is a debilitating neurological disorder impacting millions worldwide. Calcitonin Gene-Related Peptide (CGRP) has emerged as a key player in migraine pathophysiology, leading to the development of targeted therapies. This study reviews novel CGRP-targeted treatments, including monoclonal antibodies small molecule inhibitors/nutraceuticals and introduces Asarinin as a potential modulator of the pathway. Asarinin, a natural compound found in various plants, is examined for its pharmacological potential in migraine management. Pharmacokinetic assessments, toxicological modelling, molecular property analysis, and network pharmacology were conducted. Molecular docking and dynamics studies with CGRP reveal potential interactions, providing a foundation for understanding Asarinin's therapeutic effects. Asarinin's favourable pharmacokinetics, safety profile, and bioactivity, supporting its candidacy as a therapeutic agent. In-depth molecular docking studies with the CGRP receptor (PDB: 6ZHO) demonstrate strong binding affinity (− 10.3kcal/mol), while molecular dynamics simulations unveil the dynamic behavior of the Asarinin-CGRP complex, (− 10.53 kcal/mol) for Atogepant-CGRP complex. Network analysis highlights key proteins in migraine pathology, indicating Asarinin's potential efficacy. The groundwork for future investigations, suggests Asarinin as a promising candidate for migraine management by targeting OPRM1 pathway. The integration of diverse assessments provides a comprehensive understanding of Asarinin's potential and paves the way for further preclinical and clinical research.

## Introduction

Migraine is a complex neurological disorder characterized by recurrent episodes of moderate to severe headaches, often accompanied by symptoms such as nausea, sensitivity to light and sound, and visual disturbances^1–3^. This debilitating condition significantly impacts the quality of life for millions of individuals worldwide. One promising avenue of research and treatment for migraines revolves around the role of Calcitonin Gene-Related Peptide (CGRP), a neuropeptide that plays a crucial role in the pathophysiology of migraines. CGRP is involved in the dilation of blood vessels and the transmission of pain signals in the nervous system^4–6^. Its elevated levels during migraine attacks have led researchers to focus on developing therapies that target CGRP to alleviate symptoms and prevent the onset of migraine episodes. In recent years, the development of CGRP-targeted therapies has gained substantial attention and shown promising results in migraine management^7^. Common adverse effects reported include mild to moderate injection-site reactions, such as pain or erythema. Systemic reactions like fatigue, constipation, and hypersensitivity reactions have also been noted, albeit infrequently. Severe adverse events are rare but can include anaphylaxis or severe hypersensitivity. The cost of CGRP antagonists can be a significant consideration in clinical practice. These biologic therapies are typically expensive, often ranging from several thousand to tens of thousands of dollars annually per patient. Cost-effectiveness analyses have been conducted to evaluate their economic impact compared to conventional migraine treatments. Factors such as potential reductions in healthcare utilization and improved quality of life are taken into account when assessing their value. In terms of tolerability, CGRP antagonists are generally well-tolerated by patients. They offer a notable advantage over traditional migraine treatments by minimizing systemic side effects commonly associated with oral medications, such as gastrointestinal disturbances or sedation. Patients often appreciate the predictable dosing schedule of these biologics, which may improve treatment adherence and overall therapeutic outcomes. These novel treatments include monoclonal antibodies and small molecule inhibitors designed to either block CGRP receptors or inhibit the release of CGRP itself. By specifically targeting the CGRP pathway, these therapies aim to disrupt the cascade of events leading to migraine, providing patients with more effective and targeted relief^8^. The intricate relationship between migraines and CGRP, delving into the latest advancements in research, potential treatment options, and the transformative impact these developments may have on the lives of those affected by migraines. While pharmaceutical interventions are commonly used to manage migraines, there is growing interest in natural products as complementary or alternative approaches.

Natural products, derived from plants and other sources, have been explored for their potential in alleviating migraine symptoms and preventing recurrent attacks^9^. Feverfew is an herb known for its anti-inflammatory properties. Some studies suggest that it may help reduce the frequency and severity of migraines^10^. Butterbur (Petasites) has been studied for its potential in preventing migraines. It may work by reducing inflammation and stabilizing blood vessels^11,12^. Lavender oil is often used in aromatherapy to promote relaxation and alleviate stress, which can be triggers for migraines^13^. Peppermint oil has been associated with headache relief, possibly due to its muscle-relaxing and analgesic properties. Some studies suggest that magnesium supplementation may help reduce the frequency of migraines, possibly by stabilizing blood vessels and reducing cortical spreading depression^14^. Riboflavin has shown promise in reducing the frequency and duration of migraines, possibly by improving cellular energy production. Mind–body practices, such as yoga and meditation, can help manage stress, a common trigger for migraines^15,16^. These techniques promote relaxation and may contribute to overall well-being. Some individuals find relief by identifying and avoiding specific trigger foods, such as chocolate, caffeine, and certain additives^17,18^. Acupuncture, a key component of Traditional Chinese Medicine, involves the insertion of thin needles into specific points on the body. Some studies suggest that acupuncture may help reduce the frequency and intensity of migraines^19–21^. In recent years, Asarinin exhibited significant anti-inflammatory effects, which could be beneficial for various inflammatory conditions and may contribute to alleviating the inflammatory processes associated with migraine attacks. Inflammation is a key component in the pathophysiology of migraines, and substances with anti-inflammatory properties can potentially mitigate the severity and duration of migraine symptoms. Its potent antioxidant properties help in combating oxidative stress, a factor implicated in migraine pathogenesis. Its antioxidant activity^22^ helps neutralize harmful free radicals, potentially reducing oxidative damage to cells and tissues that may contribute to overall migraine prevention or attenuation. Additionally, the compound's analgesic properties provide pain relief, making it a valuable candidate for further research in pain management^23^ could be crucial in mitigating the intense pain experienced during migraine attacks. Additionally, it also exhibits neuroprotective properties, which can be beneficial for individuals experiencing migraines. This multifaceted profile of Asarinin underscores its potential as a versatile therapeutic agent and may show promise in the treatment of migraines, offering potential relief where traditional methods fall short. Migraines are associated with neuronal hyperexcitability, and neuroprotective agents like Asarinin may help safeguard nerve cells from damage during migraine episodes. The purpose of docking and dynamic studies of Asarinin with CGRP in the context of migraine research is multifaceted and aims to advance our understanding of Asarinin's potential as a therapeutic agent for migraine management. The intricate relationship between migraines and CGRP provides a foundation for investigating how Asarinin may interact with CGRP and contribute to migraine relief.

## Methodology

### Pharmacokinetic assessment

Extracting information from the PubChem server reveals the standard GRINs (Graphical Representation of the Chemical Structure) of Asarinin. Pharmacokinetic studies were completed utilizing pkCSM and Swiss ADME, with the ADMET profile retrieved from the host computer using canonical SMILES. Both pkCSM and Swiss ADME furnish details on the drug's pharmacokinetics (PK), pharmacodynamics (PD), and toxicology (Toxicity). The web-based pkCSM application facilitates the exploration of the pharmacokinetic properties of drugs, and their physicochemical properties was conducted using pkCSM software. Lipinski's rule of five, which identifies five physicochemical features influencing a molecule's efficacy, safety, or metabolism, was employed to analyze Asarinin^24,25^.

### Predicting drug bioactivity: in silico analysis with MOLINSPIRATION

The Asarinin underwent in silico testing using MOLINSPIRATION software to assess drug similarity and predict bioactivity. The likelihood of a molecule being active increases with a higher score. A chemical with a bioactivity score exceeding 0.00 is deemed to possess notable biological activities. Scores ranging between -0.50 and 0.00 on the bioactivity scale indicate high activity, whereas values falling below -0.50 are indicative of inactivity^26,27^.

### Toxicological modeling and simulation

To mitigate potential complications arising from drug withdrawal, such as organ system failure or damage, it is imperative to conduct toxicological testing. The OSIRIS property explorer software was employed, utilizing PubChem structures to assess the toxicity levels of the compounds. Substances were categorized on a color scale, indicating their potential to cause cancer, induce mutations, irritate, affect reproduction, and act as a potential drug. The computed based on specific toxicity criteria: high risk indicated in red, medium risk denoted in yellow, and low risk represented in green^28,29^.

### Molecular docking

Molecular docking is a computational technique used in structural biology, bioinformatics, and drug discovery to predict how two molecules, typically a small ligand and a larger protein interact with each other. The primary goal of molecular docking is to predict the preferred orientation and conformation of the ligand when bound to the target protein^30^.

#### Selection of CGRP protein and preparation of Asarinin ligand

Crystallographic or homology-based models of the CGRP protein were obtained from relevant databases such as the Protein Data Bank. Multiple structures, representing different conformations or states, were considered for a comprehensive analysis. The chemical structure of Asarinin was obtained from PubChem database, and its 3D structure was optimized using Chem3D Pro 12.0. The rationale behind selecting the specific PDB ID 6ZHO for the CGRP protein, related to migraine, involves several key factors. PDB ID 6ZHO represents a high-resolution crystal structure of the CGRP receptor, ensuring an accurate representation of the protein's conformation, including critical regions involved in ligand binding and receptor activation. During the ligand preparation process, we performed several optimization steps to ensure accuracy and reliability. These included energy minimizations using molecular mechanics and quantum mechanics methods, conformational analysis to explore the conformational space thoroughly, optimization of protonation states to reflect physiological pH conditions, consideration of different tautomeric forms, application of solvation models to simulate the aqueous environment, and filtering ligands using Lipinski's Rule of Five and other drug-likeness criteria. These steps collectively improved the quality and reliability of our ligand preparations, enhancing the accuracy of our in silico predictions. The ligand was prepared by assigning appropriate charges and minimizing energy to ensure a stable conformation^31–33^.

#### Molecular docking setup and validation

The binding interactions between asarinin and the CGRP receptor PDBID:6ZHO were investigated using AutoDockTool v1.5.6. Grid parameters were carefully chosen to include the ligand-binding site, considering ligand and receptor flexibility. The proteins in this study were designed, and the modified protein was optimized by stabilizing amino acid residue ionization and tautomeric states. This technique removed water molecules and added hydrogen. To keep the modified protein structure available for docking studies, a PDB file was created. This work optimized proteins using the Molegro molecular viewer to change bond order after water and covalently bound ligands were removed. The molecular mechanics force field decreased energy once charge and protonation states were assigned. AutoDock generated docking conformers by computing Gasteiger charges and assessing the ligand's rotatable bonds. The receptor grids were built from defined receptor locations, and the grid boxes were created with the receptor grid axes as the domain, centrally situating the macromolecule in the checkerboard. Maps were created using Autogrid 4. All molecular docking simulations used Lamarckian genetics. Docking involved 50 iterations, 150 subjects, 2.5 million assessments, and 27,000 generations. Biovia Discovery Studio 2021 imported docking snapshots and exported docked structures to pdbqt. The docked conformations were analyzed for asarinin-CGRP receptor binding affinity, modes, and critical interactions. Visual inspection and computational measures like binding energy and hydrogen bond analyses were used to assess complex stability using Biovia Discovery Studio 2021^34–37^.

### Molecular dynamics simulations

Molecular dynamics simulations were used to study the Asarinin-CGRP receptor complex's dynamics. Solvation was performed, and force fields were used to observe the interaction's stability and flexibility over time. This helped us understand Asarinin's long-term effects on the CGRP receptor. MD simulations were done on top-docked Asarinin and CGRP protein conformations (PDB: 6ZHO) using the Schrodinger Desmond 5.6.1 modelling software in Linux. PRODRG generated critical files, including gro and Asarinin inhibitor files, before simulation. After the MMFF94 force field, solvation, ion addition, energy minimization, and system equilibration tests (NVT and NPT) were performed. After generating trajectories with a 10-nm MD simulation, RMSD, RMSF, and radius of gyration were examined. To investigate the protein dynamics when Asarinin binds to 6ZHO, MD simulation experiments examined the docked protein's protein–ligand complex. If the original structure was energetically unstable, Desmond was critical in equilibrating the system to a stable shape. Periodic boundary conditions (PBCs) and the single-point charge (SPC) water model TIP3P were used to solve each compound in a 10-member water box. For charge neutrality, Na+ and Cl− ions were introduced to the OPLS2005 force field, which represented the protein and ligand. Energy minimization took 2000 steps, followed by a 10 ns production cycle. The system was then manufactured in the NPT ensemble, progressively heating to 300 K and steady pressure. For this, the Nose–Hoover thermostatic algorithm and Martina–Tobias–Klein method were used. Long-range electrostatic interactions were simulated using particle mesh Ewald (PME) with 0.8 grid spacing. The Desmond package's Simulation Interaction Diagram tool was used to examine ligand–protein interactions. Similar methods were used to compare protein and ligand RMSD and RMSF values to reference values. Trajectories are used in molecular dynamics modelling to anticipate molecular variability. The best-posed bound complexes with the strongest binding energies from molecular docking were individually obtained and analyzed using Schrödinger Desmond MD simulation. The drug's topology and gro file were prepared using PRODRUG (an online service) before MD simulation. The protein–ligand combination was built using the OPLS2005 force field, followed by energy minimization and ion addition. NVT and NPT were used for system equilibration after energy minimization. A leap-frog integrator with a step size of 2 fs was used to run a ten-nanosecond MD run. Data were stored every 2 picoseconds for stability study^38–40^.

### MMGBSA analysis

MMGBSA (Molecular Mechanics/Generalized Born Surface Area) is a method used to estimate the binding free energy of ligands to proteins, combining molecular mechanics energies with solvation energies for accurate predictions. The obtained trajectory file from MD simulation was subjected to the MMGBSA panel in Schrodinger Maestro 12.0 Software. Various parameters such as molecular mechanics energy (bonded, electrostatic, van der Waals interactions), solvation energy (polar solvation via the Generalized Born model), and nonpolar solvation energy (via surface area calculations), were calculated. The binding free energy (ΔG bind) is derived as the difference in free energy between the complex and the sum of the individual protein and ligand energies. Finally, the average binding free energy and its standard deviation were computed^41,42^.

### Network pharmacology

Asarinin targeted proteins were screened by the SwissADME prediction tool http://www.swisstargetprediction.ch/ with the help of specific compound SMILES. The STRING tool constructed the PPI network, Gene Ontology (GO) aspects, and KEGG pathway analysis (P < 0.01) focusing on common targets in "homo sapiens" with an interaction score threshold of 0.4 by searching “CGRP”. Cytoscape v_3.10.0 software was used by searching CGRP in protein query to analyze potential targets, and pathways to evaluate Asarinin efficacy against migraine. Network topological parameters (degree, betweenness, proximity) and primary targets were identified. Protein–Protein Interactions (PPI) were crucial and analyzed for understanding cellular mechanisms. CytoScape_v3.10.0 software was utilized to generate the network diagram and network analysis^43–48^.

## Results

### Pharmacokinetic assessment of Asarinin

Asarinin serves as a substrate for the CYP3A4 enzyme and acts as an inhibitor for CYP1A2, CYP2C9, CYP2C19, and CYP3A4. Its clearance was determined to be − 0.126 ml/min/kg. According to the pkCSM database, the maximum safe daily intake for Asarinin in humans is reported to be 0.089 mg/kg, with a predicted LD_50_ of 2.833 mol/kg. Table 1 provides a comprehensive overview of the pharmacokinetic profile of Asarinin, including its substrate and inhibitory actions on various cytochrome P450 enzymes, clearance, maximum safe daily intake, and LD_50_ predictions. Notably, Asarinin does not induce skin sensitivity, liver damage, or renal damage. It achieves a favourable bioactivity score for therapeutic targets, establishing the reliability of this pharmacokinetic technique in drug development and analysis. SwissADME analysis of Asarinin's pharmacokinetic profile suggests high gastrointestinal absorption and identification as a P-glycoprotein I inhibitor. While Asarinin exhibits moderate absorption and distribution throughout the body, its intestinal absorption rate of 97.81% stands out significantly. The permeability of Asarinin through the blood–brain barrier is calculated to be − 0.862 (log BB), indicating effective passage, while its central nervous system permeability is − 2.939 (log PS), signifying enhanced penetration and the potential for pharmacological effects in the CNS. ADMET predictions underscore Asarinin's notable pharmacological properties, particularly its role as a P-glycoprotein I inhibitor, as evidenced by absorption properties below the threshold values. The physiochemical profile of Asarinin is appealing, with anticipated values falling within the acceptable range.

**Table 1:** Pharmacokinetic profile of Asarinin.

Property	Model name	Predicted value
Absorption	Water solubility	− 4.223 log mol/L
Absorption	Caco2 permeability	1.399 log Papp in 10–6 cm/s
Absorption	Intestinal absorption (human)	97.81%
Absorption	Skin permeability	− 2.772 log Kp
Absorption	P-Glycoprotein substrate	No
Absorption	P-Glycoprotein I inhibitor	Yes
Absorption	P-Glycoprotein II inhibitor	No
Distribution	VDss (human)	− 0.17 log L/kg
Distribution	Fraction unbound (human)	0 Fu
Distribution	BBB permeability	− 0.862 log BB
Distribution	CNS permeability	− 2.939 log PS
Metabolism	CYP2D6 substrate	No
Metabolism	CYP3A4 substrate	Yes
Metabolism	CYP1A2 inhibitior	Yes
Metabolism	CYP2C19 inhibitior	Yes
Metabolism	CYP2C9 inhibitior	Yes
Metabolism	CYP2D6 inhibitior	No
Metabolism	CYP3A4 inhibitior	Yes
Excretion	Total clearance	− 0.126 log ml/min/kg
Excretion	Renal OCT2 substrate	No
Toxicity	AMES toxicity	Yes
Toxicity	Max. tolerated dose (human)	0.089 log mg/kg/day
Toxicity	hERG I inhibitor	No
Toxicity	hERG II inhibitor	No
Toxicity	Oral rat acute toxicity (LD50)	2.883 mol/kg
Toxicity	Oral rat chronic toxicity (LOAEL)	1.568 log mg/kg_bw/day
Toxicity	Hepatotoxicity	No
Toxicity	Skin sensitisation	No
Toxicity	*T. pyriformis* toxicity	0.34 log ug/L
Toxicity	Minnow toxicity	0.039 log mM

### Toxicological assessment of Asarinin

**Fig. 1:**
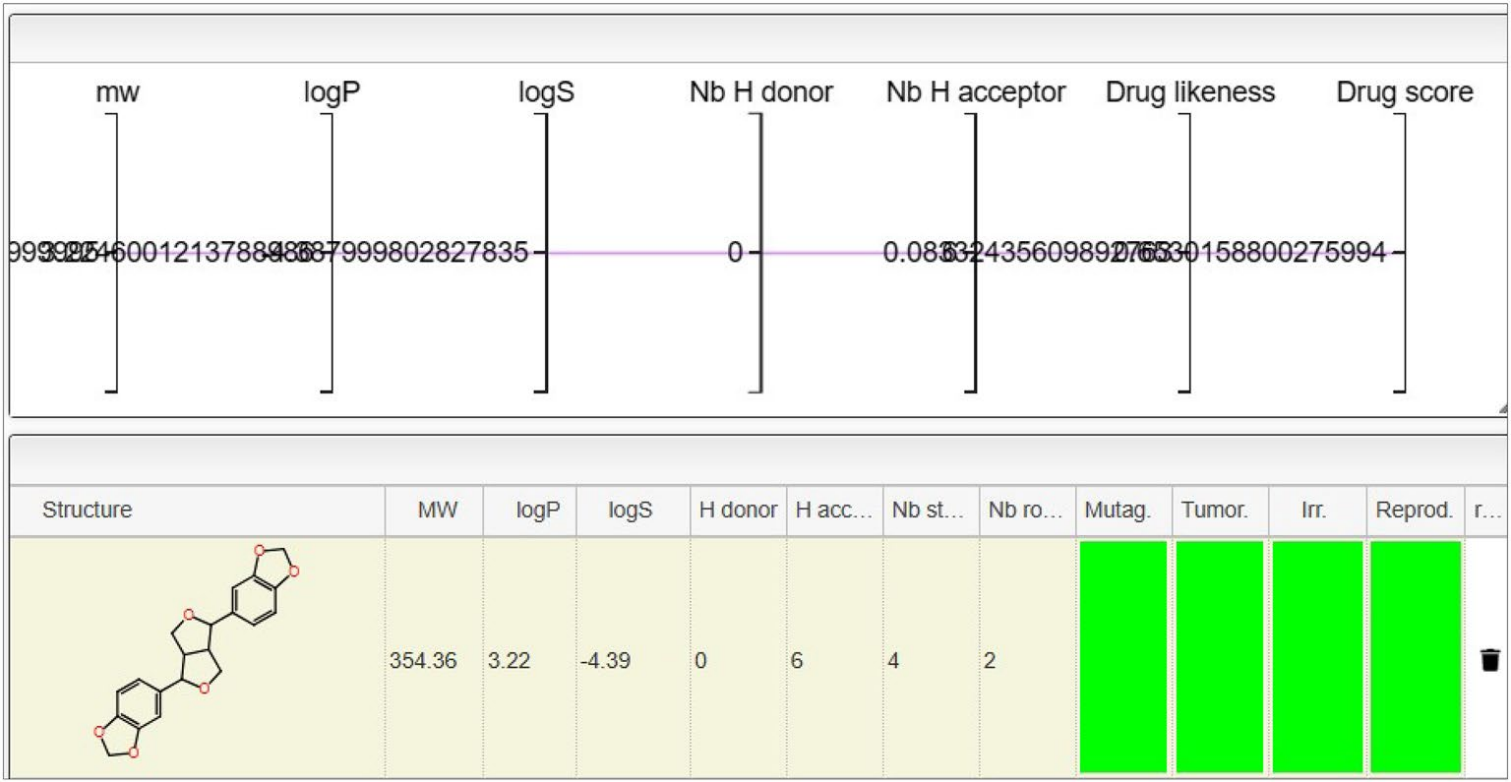
The Asarinin compound is moderately lipophilic, as indicated by its logP value of 3.22 and molecular weight of 354.36. Despite its low solubility in water (logS of − 4.39), its interactions with biological targets may be impacted by the fact that it lacks hydrogen bond donors and contains six hydrogen bond acceptors. The presence of two stereocenters and four rotatable bonds suggests that the molecule is quite flexible. There is little to no concern for carcinogenicity, irritating potential, reproductive toxicity, or tumorigenicity, as all of the toxicity forecasts are green. In general, the chemical shows promise as a potential drug development candidate due to its low projected toxicity, acceptable drug-likeness, and drug score.

The toxicity of the examined inhibitors was determined through in-silico methods, and the substance's physicochemical properties were assessed using the OSIRIS Property Explorer illustrated in Fig. 1. It is imperative to scrutinize the toxicity profile of Asarinin before progressing to clinical trials. Asarinin demonstrated a lack of mutagenic, carcinogenic, irritant, or reproductive effects, aligning with the toxicity characteristics of the tested compounds. The majority of proposed inhibitors exhibited acceptable toxicity profiles, rendering them plausible therapeutic alternatives. The OSIRIS Property Explorer endorsed the substance with a clean health bill regarding the mentioned aspects, enhancing its safety. The Topological Polar Surface Area (TPSA) of a molecule is a critical determinant of its absorption and distribution within the body. OSIRIS Property Explorer computed Asarinin's TPSA to be 55.38. The recorded drug-likeness score for this molecule was 0.65. A positive result indicates that the molecule shares common features with commercially available drugs. The essence of a drug, derived from the combination of its molecular weight, toxicity risks, and cLogP, is crucial in determining its suitability for a given treatment. Asarinin's drug score was determined to be 0.65.

### Bioactivity Score Assessment of Asarinin

The Molinspiration online tool extends complimentary services to the internet chemistry community, encompassing the prediction of bioactivity scores for crucial drug targets such as GPCR ligands, ion channel modulators, kinase inhibitors, and nuclear receptors. Additionally, it facilitates the computation of essential molecular properties, including polar surface area, log P, and the count of hydrogen bond donors and acceptors, among others. Table 2 displays the bioactivity scores for Asarinin across crucial drug targets, as predicted by the Molinspiration online tool. The absence of PAINS warnings is also highlighted. Furthermore, the tool delves into medicinal chemistry aspects such as synthetic accessibility and the presence of PAINS warnings. Notably, none of the synthesized chemicals elicited a warning, as indicated by a PAINS alert value of 0.

**Table 2:** Bioactivity score and physiochemical parameters of Asarinin.

Bioactivity score		Physiochemical parameters	
GPCR ligand	0.02	cLogP	3.22
Ion channel modulator	− 0.31	TPSA	55.38
Kinase inhibitor	− 0.27	Drug Likeliness	0.08
Nuclear receptor ligand	− 0.09	HBA	6
Protease inhibitor	− 0.15	HBD	0
Enzyme inhibitor	0.03	Nb stereocenters	4
		Drug score	0.65

### Docking assessment and validation

**Fig. 2:**
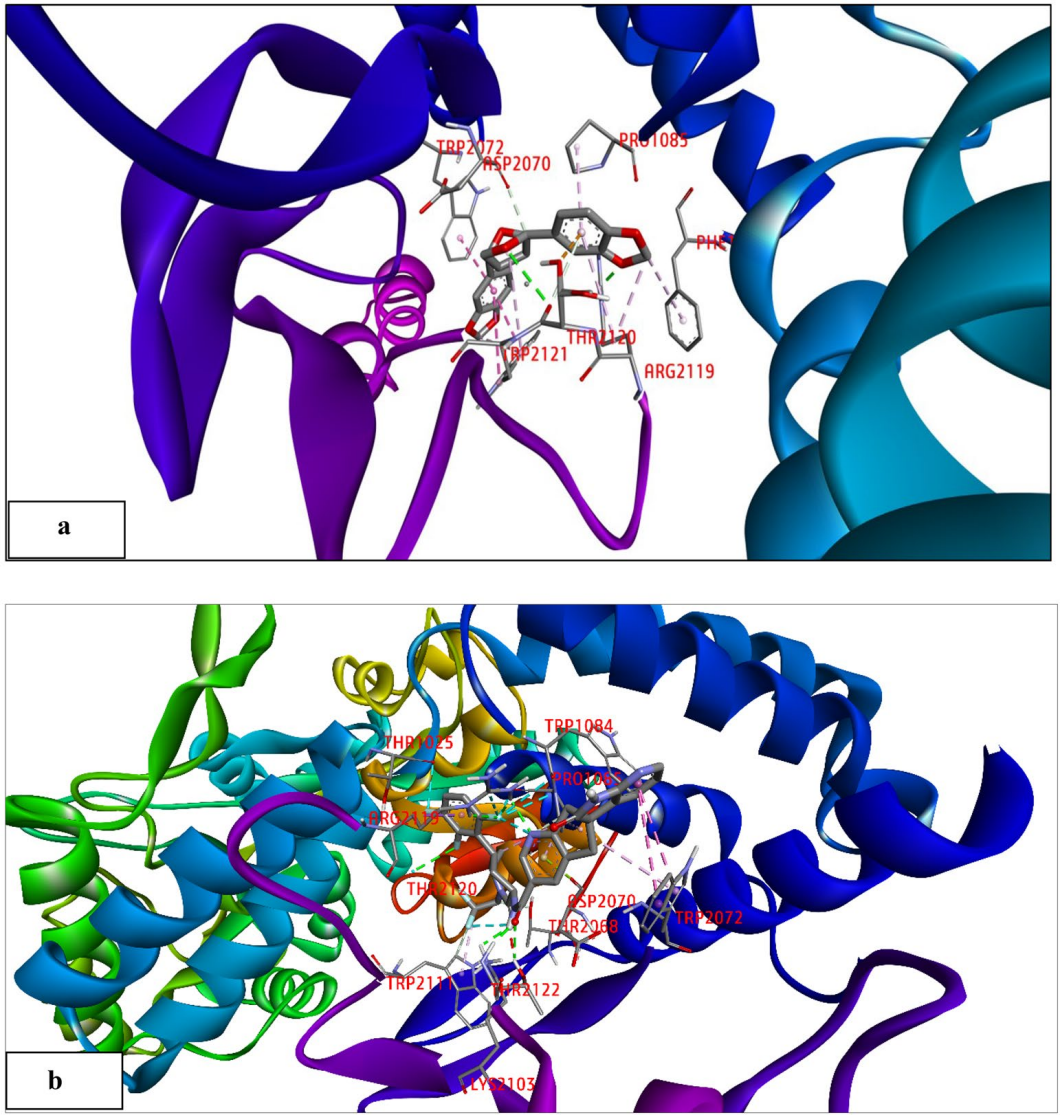
This figure shows a snapshot of a molecular docking of a ligand's interaction with a protein's binding site. Ribbons in purple and blue depict several secondary structural components of the protein. In the middle of the stick figure is the ligand, which is colored according to its type of atom. Important residues that interact with each other are highlighted in red. These include TRP2072, ASP2070, PRO1085, THR2120, and ARG2119. The interactions are shown by the dashed lines, which vary in color from purple for hydrogen bonds to green for hydrophobic or π–π stacking interactions. (**a**) Presents the molecular docking analysis of Asarinin with CGRP (PDB: 6ZHO), revealing a hydrogen bond formation with specific amino acid residues (LYS2103, ARG2119, THR2120, THR2122). Binding affinity = − 10.03 kcal/mol. (**b**) Presents the molecular docking analysis of Atogepant with CGRP (PDB: 6ZHO), revealing a hydrogen bond formation with specific amino acid residues (LYS2103, ARG2119, THR2120, THR2122). Binding affinity = − 10.53 kcal/mol. The ligand's possible effectiveness as a medication candidate is dependent on its strong and stable binding affinity, which is suggested by the presence of numerous hydrogen bonds and hydrophobic interactions.

Among various PDB IDs examined, we identified 6ZHO as the most suitable candidate for further docking analysis, supported by its commendable Ramachandran plot, indicating the structural integrity necessary for robust molecular docking studies. Asarinin showed the almost equal binding energy in CGRP (PDB: 6ZHO = − 10.03 kcal/mol) when compared with Atogepant (PDB: 6ZHO = − 10.53 kcal/mol). Since molecular docking persists as a computer simulation approach used for determining the shape of a receptor ligand complex, the hypothesis desires to be tested empirically in future studies, but the data presented here suggests that Asarinin might have a significant ability to directly connect with CGRP. The Asarinin and Atogepant formed a hydrogen bond with amino acid residues LYS2103, ARG2119, THR2120, and THR2122 was illustrated in Fig. 2 and Table 3 details the hydrogen bonding interactions between Asarinin and amino acid residues in the CGRP (PDB: 6ZHO) receptor. The distances of the hydrogen bonds are presented for each interaction. In most selected targets, a considerably increased number of amino acid residues engaged in hydrogen bonding and van der Waals interactions was related with shorter hydrogen bond lengths (less than 3.0). The docking findings for Asarinin and Atogepant, with binding affinities were corroborated by a thorough examination of their binding poses and important interactions inside the target protein's active region which demonstrate the docking protocol's reliability and precision. Therefore, Asarinin had significant hydrogen bonds and hydrophobic interactions with important residues, which were similar to those seen with Atogepant. The protocol's ability to estimate ligand–protein binding affinities is validated by its concordance with experimental results.

**Table 3:** The conventional hydrogen bond interaction and their distance between of Ligand (Asarinin & Atogepant) with CGRP (6ZHO) complex.

Index	Residue	Amino acid	Distance H–A	Distance D–A	Donor angle	Protein donor	Side chain
Asarinin							
1	2103A	LYS	3.15	3.74	117.9	Yes	Yes
2	2119A	ARG	1.88	2.73	138.55	Yes	Yes
3	2119A	ARG	2.75	3.4	122.15	Yes	Yes
4	2120A	THR	3.18	4.08	158.18	Yes	Yes
5	2122A	THR	3.13	3.86	129.68	Yes	No
6	2122A	THR	2.94	3.8	152.58	Yes	Yes
Atogepant							
1	2103A	LYS	2.87	3.46	117.62	Yes	Yes
2	2119A	ARG	3.29	3.62	100.85	Yes	Yes
3	2119A	ARG	2.11	2.53	102.01	Yes	Yes
4	2120A	THR	3.20	4.09	156.79	Yes	Yes
5	2122A	THR	2.76	3.61	148.80	Yes	Yes
6	2122A	THR	2.82	3.61	133.77	No	Yes
7	2122A	THR	1.60	2.54	151.03	Yes	No

*AA* amino acids, *H–A* distance between hydrogen and acceptor atoms, *D–A* distance between donor and acceptor atoms.

### Molecular dynamic assessment

**Fig. 3:**
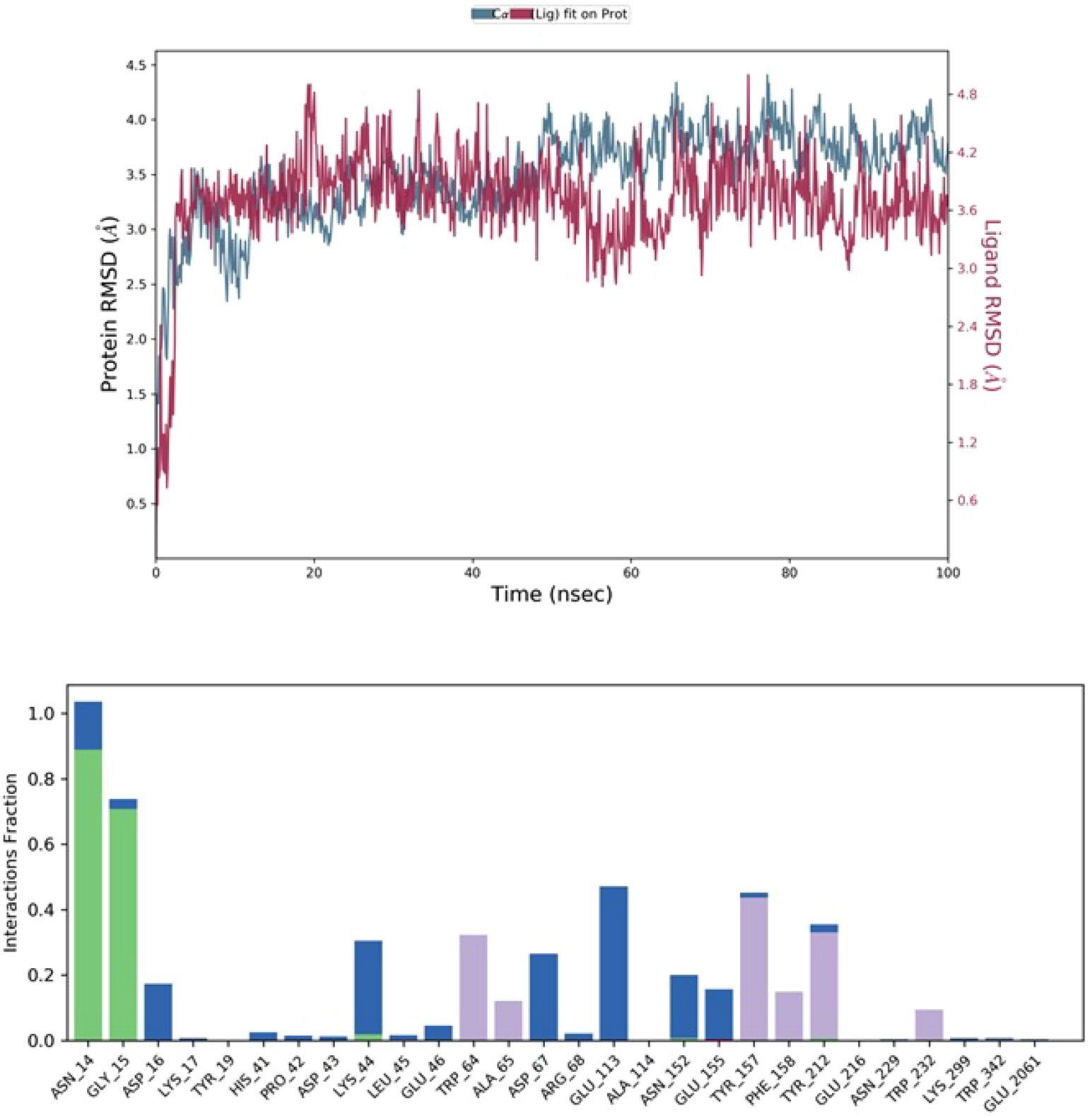
The stability of the complex's protein and ligand, as simulated by a 100-ns molecular dynamics run, is illustrated in the image. Root Mean Square Deviation (RMSD) plots of the protein (blue line) and ligand (red line) are displayed in the top panel. After an initial period of rise, the protein RMSD stabilizes at approximately 3–4 Å, suggesting that it attains a relatively stable shape. This indicates that there are no major changes to the protein structure during the simulation. Likewise, the ligand RMSD varies within the range of 3–4 Å, showing that the ligand maintains a constant binding position in relation to the protein. Since the ligand RMSD remains relatively constant throughout the simulation, it is safe to assume that it maintains a stable contact with the protein and does not undergo any substantial dissociation or changes to its binding conformation. Taking all of these findings into account, it appears that the protein and ligand both keep their conformations steady, suggesting that the protein–ligand combination remains stable during the simulation.

**Fig. 4:**
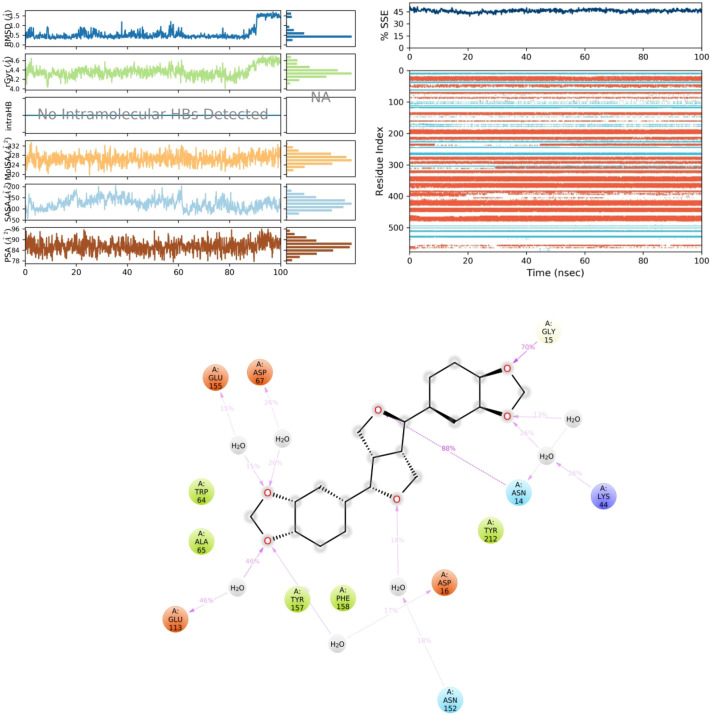
Depicts the 2D critical geometry of Asarinin with 6ZHO post-molecular dynamics simulation. Additionally, it presents graphs illustrating the composition of secondary structure elements (SSE) for each orbital frame throughout the simulation. Each amino acid is represented by a coloured circle with its corresponding code and sequence number. Here we may see the ligand-amino acid or water-molecule interactions graphically represented by dotted lines and arrows with percentages next to them. The ligand-ASP 67 (26%), ASN 14 (88%), GLU 113 (46%), LYS 44 (26%), GLY 15 (70%), and GLU 155 (15%) interactions. The bonding likelihood varies, with ASN 14 showing the highest interaction probability. Shown are both direct and water-mediated interactions, illustrating how dynamic and complicated the binding environment is. To comprehend the possible therapeutic uses of the ligand, it is essential to grasp the binding mechanism, and this comprehensive map of ligand–amino acid interactions, particularly the highlighted percentages, gives important insights in this regard.

Molecular dynamics simulations were carried out on the Asarinin and 6ZHO docked complexes. In the early phases, the root mean square deviation (RMSD) showed a correlation with the ligand RMSD (Asarinin) and persisted for up to 100 ns, suggesting the occurrence of multiple interactions with amino acid residues. The accompanying visual figure summarizes these interactions and the number of touchpoints. Key amino acids, such as ASN14, GLY15, and LYS44 were highlighted for their essential role in establishing interactions and contributing to the non-competitive binding site, as depicted in Fig. 3. After MD simulation, the 2D critical geometry of Asarinin with 6ZHO revealed varied interactions with the optimal binding site of 6ZHO. Graphs illustrating the composition of secondary structure elements (SSE) for each orbital frame throughout the simulation were provided, and the lower chart in Fig. 4 depicted alterations in SSE assignment for each residue over time.

### MMGBSA analysis

The free energy calculations provide insight into the binding affinities of Asarinin and Atogepant. Asarinin exhibits a more negative total binding free energy (Δ*G* Bind =  − 39.4678 kcal/mol) compared to Atogepant (Δ*G* Bind =  − 34.84066 kcal/mol), indicating that Asarinin has a stronger binding affinity to the target. This is an important factor in drug design as a more negative binding free energy generally correlates with higher efficacy. Examining the individual energy contributions, Atogepant demonstrates stronger lipophilic interactions (Δ*G* Bind_Lipo =  − 26.90095 kcal/mol) and Asarinin (Δ*G* Bind_Lipo =  − 22.5455 kcal/mol); van der Waals forces (Δ*G* Bind_vdW =  − 40.94962 kcal/mol) compared to Asarinin (Δ*G* Bind_vdW =  − 39.1126 kcal/mol). Atogepant also shows a slightly stronger hydrogen bonding contribution on comparison with Asarinin (Δ*G* Bind_Hbond =  − 1.281017 kcal/mol and Δ*G* Bind_Hbond = − 0.31806 kcal/mol). However, these favourable interactions are offset by Atogepant's (Δ*G* Bind_Coulomb = − 1.351845 kcal/mol), higher ligand strain energy (15.564371 kcal/mol) and less favourable solvation energy (Δ*G* Bind_Solv_GB = 25.209839 kcal/mol), suggesting that Atogepant undergoes more structural deformation and experiences greater desolvation penalties upon binding. In contrast, Asarinin, while having an unfavourable electrostatic contribution (Δ*G* Bind_Coulomb = 2.255693 kcal/mol), benefits from significantly lower ligand strain energy (5.749303 kcal/mol) and less unfavourable solvation energy (Δ*G* Bind_Solv_GB = 16.3162 kcal/mol). These factors contribute to its overall more favourable binding energy. Additionally, the lower ligand efficiency value for Asarinin (− 1.51799) compared to Atogepant (− 0.810248) was listed in Table 4. It suggests that Asarinin is less efficient in binding per non-hydrogen atom, but the overall binding energy remains more favourable. In summary, while Atogepant has strong individual interactions, its overall binding is less favourable due to higher strain and solvation penalties. Asarinin’s lower strain and solvation penalties contribute to its stronger binding affinity, making it potentially a more effective binder to the target.

**Table 4:** Comparative free energy calculations and binding affinities of Atogepant and Asarinin.

	Atogepant	Asarinin
Molecular docking		
Binding energy	− 10.53 kcal/mol	− 10.03 kcal/mol
MMGBSA		
r_psp_MMGBSA_dG_Bind	− 34.84066	− 39.4678
r_psp_MMGBSA_dG_Bind_Coulomb	− 1.351845	2.255693
r_psp_MMGBSA_dG_Bind_Covalent	11.635876	4.605373
r_psp_MMGBSA_dG_Bind_Hbond	− 1.281017	− 0.31806
r_psp_MMGBSA_dG_Bind_Lipo	− 26.90095	− 22.5455
r_psp_MMGBSA_dG_Bind_Solv_GB	25.209839	16.3162
r_psp_MMGBSA_dG_Bind_vdW	− 40.94962	− 39.1126
r_psp_Lig_Strain_Energy	15.564371	5.749303
r_psp_Prime_MMGBSA_ligand_efficiency	− 0.810248	− 1.51799

### Network analysis

**Fig. 5:**
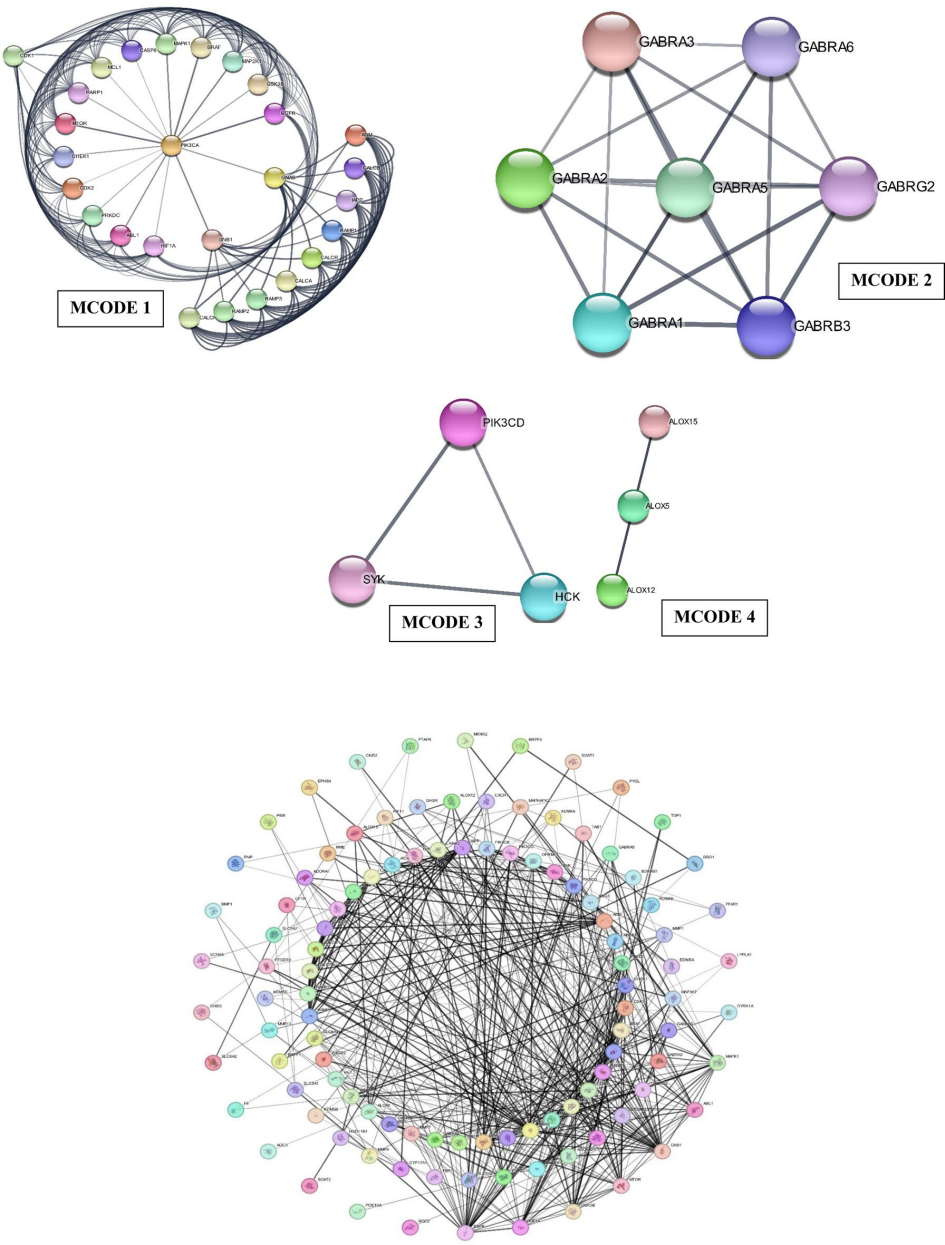
The figure shows a cell's protein–protein interaction (PPI) network. Visually represents the results of Gene Ontology (GO) and KEGG pathway analyses based on the PPI network. OPRM1, GNB1, GNAS, and other key targets are identified, shedding light on potential pathways implicated in migraine with aura. PPI network based on cluster analysis using the MCODE plugin. MCODE1: Proteins like PIK3CA, MTOR, and EGFR are labeled at each node. The node edges show protein interactions. Nodes with high connection, such as PIK3CA, are crucial to the network. To help visualize their biological importance, the colors of the nodes probably signify distinct functional groups or routes. Some of the most important proteins in this network include EGFR, which plays a key role in cell formation and differentiation, and MTOR, which controls cell metabolism and growth. The development of better therapeutic treatments and a better understanding of the molecular mechanisms driving migraines and similar diseases depend on our ability to decipher these interactions. MCODE 2: An important component in migraine pathogenesis is the GABA-A receptor, which consists of several subunits including GABRA1, GABRA2, GABRA3, GABRA5, GABRA6, GABRB3, and GABRG2. In this complex network, the individual subunits are shown as nodes and the connections between them are shown as edges. The gamma (GABRG), alpha (GABRA), and beta (GABRB) subunits can be more clearly distinguished by using color labeling. The intricate structure and possible interactions of the GABA-A receptor are shown in this detailed map. It emphasizes the receptor's function in inhibiting neurons and its connection to the processes of migraines. MCODE 3: The proteins PIK3CD, SYK, and HCK may play important roles in inflammation and immune cell activity. These proteins may play a role in the inflammatory pathways that contribute to the pathophysiology of migraines, which have been linked to neuroinflammatory processes. Exploring the interplay between PIK3CD, SYK, and HCK may lead to the discovery of new molecular targets for the improvement of migraine treatments, with an emphasis on regulating inflammatory responses and immunological responses. MCODE 4: Proteins like ALOX15, ALOX5, and ALOX12 are essential for the inflammatory response and bioactive lipid mediator synthesis; they are involved in the metabolism of arachidonic acid. Fatty acid metabolism impacts inflammation and vascular functions; ALOX12 is engaged in this process; ALOX5 leads to the generation of leukotrienes; and ALOX15 metabolizes arachidonic acid to create lipid mediators. Due to inflammation's central role in migraine pathophysiology, these interactions take on added significance when considering migraines. We have utilized KEGG analysis for the respective gene annotation and utilized Cytoscape_v1.10.0 to create this figure.

To delve deeper into the complexities of PPI networks, we inputted data on the 42 common targets into the STRING database. This led to the creation of a network with 42 proteins as nodes connected by 223 PPI relationships, visually depicted in Fig. 5. The graphical representation of protein interactions was accomplished using Cytoscape v_3.10.0. After establishing the PPI network, we utilized the network analyzer to calculate degree values. The analysis focused on Gene Ontology (GO) and KEGG pathways, yielding 594 GO enrichment outcomes, including cellular component analysis (39), biological process analysis (519), and molecular function analysis (36). Results related to the top ten GO characteristics were specifically extracted and visualized using a bioinformatics platform. Regarding KEGG analysis, a total of 86 pathways were identified, emphasizing the gene ontology of the top ten targets, which were subsequently inputted into the bioinformatics platform. We employed the degree centrality method within Cytoscape, a widely acknowledged tool for identifying highly interconnected nodes, which often serve as critical targets or hubs in biological networks such as OPRM1, GNB1, GNAS, RAMP1, RAMP2, RAMP3, CALCR, CALCB, ADM, IAPP, and SLC5A2 are serve as central hubs within the PPI network, crucial in connecting various nodes addressing migraine with aura. The Table 5 presents results from 86 KEGG pathway enrichment analysis, highlighting pathways potentially relevant to migraine. Each pathway is characterized by observed gene counts, background gene counts, statistical strength (False Discovery Rate, FDR), and specific proteins identified within the network. Key pathways such as "Neuroactive ligand–receptor interaction", "Serotonergic synapse", "cAMP signaling pathway", and others suggest involvement in neurochemical processes and signaling mechanisms that could influence migraine pathophysiology. Genes identified within these pathways, including GABRA, GNAS, ADORA, and others, are known to modulate neurotransmitter release, vascular tone, and neuronal excitability, all critical factors in migraine development and propagation.

**Table 5:** The KEGG pathway enrichment analysis of common genes.

S. no.	#Term ID	Term description/pathway	Observed gene count	Background gene count	Strength	False discovery rate	Matching proteins in your network (labels)
1	hsa04080	Neuroactive ligand–receptor interaction	21	329	1.07	8.76E−14	BDKRB1, IAPP, GHSR, GABRA6, GABRB3, EDNRA, CALCA, PTGER3, CALCR, ADORA1, GABRA3, CNR2, CALCRL, GABRA5, GABRA1, OPRM1, CALCB, ADM, PTAFR, ADORA2A, KNG1
2	hsa04726	Serotonergic synapse	13	108	1.34	1.21E−11	MAPK1, ALOX12, CYP2C9, SLC6A4, APP, ALOX15, MAP2K1, GABRB3, GNAS, CYP2C19, ALOX5, GNB1, BRAF
3	hsa04024	cAMP signaling pathway	15	207	1.12	7.67E−11	CFTR, MAPK1, GHSR, PIK3CA, MAP2K1, EDNRA, PTGER3, PDE10A, ADORA1, GNAS, PIK3CD, MAPK9, BRAF, ADORA2A, PIK3CB
4	hsa04270	Vascular smooth muscle contraction	13	132	1.25	7.67E−11	MAPK1, RAMP3, RAMP2, RAMP1, MAP2K1, EDNRA, CALCA, GNAS, CALCRL, BRAF, CALCB, ADM, ADORA2A
5	hsa04012	ErbB signaling pathway	11	81	1.39	1.34E−10	MAPK1, PIK3CA, EGFR, MAP2K1, GSK3B, MTOR, ABL1, PIK3CD, MAPK9, BRAF, PIK3CB
6	hsa04910	Insulin signaling pathway	12	132	1.22	7.86E−10	MAPK1, PYGL, MKNK2, PIK3CA, MAP2K1, GSK3B, MTOR, PIK3CD, MAPK9, FBP1, BRAF, PIK3CB
7	hsa04072	Phospholipase D signaling pathway	12	147	1.17	2.27E−09	MAPK1, PIK3CA, EGFR, CXCR1, MAP2K1, CXCR2, MTOR, GNAS, SYK, PIK3CD, PIK3CG, PIK3CB
8	hsa05032	Morphine addiction	10	88	1.32	4.00E−09	GABRA6, GABRB3, PDE10A, ADORA1, GABRA3, GNAS, GNB1, GABRA5, GABRA1, OPRM1
9	hsa04926	Relaxin signaling pathway	11	126	1.2	5.82E−09	MAPK1, MMP13, PIK3CA, EGFR, MAP2K1, MMP1, GNAS, PIK3CD, GNB1, MAPK9 , PIK3CB
10	hsa01522	Endocrine resistance	10	94	1.29	6.21E−09	MAPK1, PIK3CA, EGFR, MAP2K1, MTOR, GNAS, PIK3CD, MAPK9, BRAF, PIK3CB
11	hsa04062	Chemokine signaling pathway	12	186	1.07	1.44E−08	MAPK1, PIK3CA, CXCR1, MAP2K1, CXCR2, GSK3B, HCK, PIK3CD, GNB1, BRAF, PIK3CG, PIK3CB
12	hsa01521	EGFR tyrosine kinase inhibitor resistance	9	77	1.33	1.55E−08	MAPK1, PIK3CA, EGFR, MAP2K1, GSK3B, MTOR, PIK3CD, BRAF, PIK3CB
13	hsa04722	Neurotrophin signaling pathway	10	112	1.21	1.84E−08	MAPK1, PIK3CA, MAP2K1, GSK3B, MAPKAPK2, ABL1, PIK3CD, MAPK9, BRAF, PIK3CB
14	hsa04935	Growth hormone synthesis, secretion and action	10	117	1.19	2.62E−08	MAPK1, GHSR, PIK3CA, MAP2K1, GSK3B, MTOR, GNAS, PIK3CD, MAPK9, PIK3CB
15	hsa04914	Progesterone-mediated oocyte maturation	9	95	1.24	5.80E−08	MAPK1, PIK3CA, CDK2, MAP2K1, PIK3CD, CDK1, MAPK9, BRAF, PIK3CB
16	hsa04664	Fc epsilon RI signaling pathway	8	65	1.35	6.13E−08	MAPK1, PIK3CA, MAP2K1, ALOX5, SYK, PIK3CD, MAPK9, PIK3CB
17	hsa04620	Toll-like receptor signaling pathway	9	100	1.22	7.90E−08	MAPK1, TAB1, PIK3CA, MAP2K1, CASP8, MAP3K7, PIK3CD, MAPK9, PIK3CB
18	hsa04917	Prolactin signaling pathway	8	68	1.33	7.90E−08	MAPK1, PIK3CA, MAP2K1, GSK3B, CYP17A1, PIK3CD, MAPK9, PIK3CB
19	hsa04066	HIF-1 signaling pathway	9	102	1.21	8.78E−08	MAPK1, MKNK2, PIK3CA, EGFR, MAP2K1, MTOR, PIK3CD, HIF1A, PIK3CB
20	hsa04218	Cellular senescence	10	150	1.08	1.51E−07	MAPK1, PIK3CA, CDK2, MAP2K1, MTOR, MAPKAPK2, PIK3CD, CDK1, CHEK1, PIK3CB
21	hsa04668	TNF signaling pathway	9	111	1.17	1.61E−07	MAPK1, TAB1, PIK3CA, MAP2K1, CASP8, MAP3K7, PIK3CD, MAPK9, PIK3CB
22	hsa04750	Inflammatory mediator regulation of TRP channels	8	92	1.2	5.64E−07	BDKRB1, ALOX12, PIK3CA, GNAS, PIK3CD, MAPK9, KNG1, PIK3CB
23	hsa04151	PI3K-Akt signaling pathway	13	349	0.83	6.59E−07	MAPK1, PIK3CA, CDK2, , , EGFR, MAP2K1, GSK3B, MTOR, MCL1, SYK, PIK3CD, GNB1, PIK3CG, PIK3CB
24	hsa04660	T cell receptor signaling pathway	8	100	1.16	8.88E−07	MAPK1, PIK3CA, MAP2K1, GSK3B, MAP3K7, PIK3CD, MAPK9, PIK3CB
25	hsa04625	C-type lectin receptor signaling pathway	8	101	1.16	9.35E−07	MAPK1, PIK3CA, CASP8, MAPKAPK2, SYK, PIK3CD, MAPK9, PIK3CB
26	hsa04071	Sphingolipid signaling pathway	8	116	1.1	2.20E−06	MAPK1, PIK3CA, MAP2K1, ADORA1, PIK3CD, MAPK9, KNG1, PIK3CB
27	hsa04662	B cell receptor signaling pathway	7	78	1.21	2.26E−06	MAPK1, PIK3CA, MAP2K1, GSK3B, SYK, PIK3CD, PIK3CB
28	hsa04919	Thyroid hormone signaling pathway	8	120	1.08	2.67E−06	MAPK1, PIK3CA, MAP2K1, GSK3B, MTOR, PIK3CD, HIF1A, PIK3CB
29	hsa04923	Regulation of lipolysis in adipocytes	6	54	1.31	4.73E−06	PIK3CA, PTGER3, ADORA1, GNAS, PIK3CD, PIK3CB
30	hsa04666	Fc gamma R-mediated phagocytosis	7	90	1.15	5.22E−06	MAPK1 , PIK3CA, MAP2K1 , SYK, HCK, PIK3CD, PIK3CB
31	hsa04915	Estrogen signaling pathway	8	133	1.04	5.32E−06	MAPK1 , PIK3CA, EGFR, MAP2K1 , GNAS, PIK3CD, OPRM1, PIK3CB
32	hsa04370	VEGF signaling pathway	6	56	1.29	5.43E−06	MAPK1 , PIK3CA, MAP2K1 , MAPKAPK2, PIK3CD, PIK3CB
33	hsa04657	IL-17 signaling pathway	7	91	1.15	5.43E−06	MAPK1 , MMP13, MMP1, GSK3B, CASP8, MAP3K7 , MAPK9
34	hsa05169	Epstein-Barr virus infection	9	192	0.93	7.64E−06	TAB1, PIK3CA, CDK2, CASP8, MAP3K7 , SYK, PIK3CD, MAPK9 , PIK3CB
35	hsa04723	Retrograde endocannabinoid signaling	8	142	1.01	8.00E−06	MAPK1 , GABRA6, GABRB3, GABRA3, GNB1, GABRA5, GABRA1, MAPK9
36	hsa04015	Rap1 signaling pathway	9	201	0.91	1.02E−05	MAPK1 , PIK3CA, EGFR, MAP2K1 , GNAS, PIK3CD, BRAF, ADORA2A, PIK3CB
37	hsa05145	Toxoplasmosis	7	103	1.09	1.06E−05	MAPK1 , TAB1, CASP8, MAP3K7 , ALOX5, MAPK9 , PIK3CG
38	hsa04150	mTOR signaling pathway	8	150	0.99	1.09E−05	MAPK1 , PIK3CA, MAP2K1 , GSK3B, MTOR, PIK3CD, BRAF, PIK3CB
39	hsa04931	Insulin resistance	7	106	1.08	1.23E−05	PYGL, PIK3CA, GSK3B, MTOR, PIK3CD, MAPK9 , PIK3CB
40	hsa04810	Regulation of actin cytoskeleton	9	209	0.9	1.30E−05	MAPK1 , BDKRB1, PIK3CA, EGFR, MAP2K1 , PIK3CD, BRAF, KNG1, PIK3CB
41	hsa04014	Ras signaling pathway	9	225	0.86	2.18E−05	MAPK1 , PIK3CA, EGFR, MAP2K1 , ABL1, PIK3CD, GNB1, MAPK9 , PIK3CB
42	hsa04110	Cell cycle	7	120	1.03	2.43E−05	CDK2, PRKDC, GSK3B, ABL1, CDK1, CHEK1, WEE1
43	hsa04152	AMPK signaling pathway	7	120	1.03	2.43E−05	CFTR, PIK3CA, MTOR, MAP3K7 , PIK3CD, FBP1, PIK3CB
44	hsa04650	Natural killer cell mediated cytotoxicity	7	120	1.03	2.43E−05	MAPK1 , PIK3CA, MAP2K1 , SYK, PIK3CD, BRAF, , PIK3CB
45	hsa04727	GABAergic synapse	6	85	1.11	3.95E−05	GABRA6, GABRB3, GABRA3, GNB1, GABRA5, GABRA1
46	hsa00590	Arachidonic acid metabolism	5	60	1.18	0.0001	ALOX12, CYP2C9, ALOX15, CYP2C19, ALOX5
47	hsa00591	Linoleic acid metabolism	4	29	1.4	0.00011	CYP2C9, ALOX15, CYP2C19, CYP3A4
48	hsa04928	Parathyroid hormone synthesis, secretion and action	6	104	1.02	0.00011	MAPK1 , MMP13, EGFR, MAP2K1 , GNAS, BRAF
49	hsa04010	MAPK signaling pathway	9	286	0.76	0.00012	MAPK1 , TAB1, MKNK2, EGFR, MAP2K1, MAPKAPK2, MAP3K7, MAPK9, BRAF
50	hsa04630	JAK-STAT signaling pathway	7	158	0.91	0.00012	PIK3CA, EGFR, MTOR, MCL1, PIM1, PIK3CD, PIK3CB
51	hsa04929	GnRH secretion	5	63	1.16	0.00012	MAPK1, PIK3CA, MAP2K1, PIK3CD, PIK3CB
52	hsa01524	Platinum drug resistance	5	70	1.11	0.00019	MAPK1, PIK3CA, CASP8, PIK3CD, PIK3CB
53	hsa04360	Axon guidance	7	176	0.86	0.00022	MAPK1, PIK3CA, GSK3B, EPHB4, ABL1, PIK3CD, PIK3CB
54	hsa04020	Calcium signaling pathway	7	191	0.83	0.00034	BDKRB1, EGFR, EDNRA, PTGER3, GNAS, PTAFR, ADORA2A
55	hsa04973	Carbohydrate digestion and absorption	4	44	1.22	0.00043	PIK3CA, SLC5A1, PIK3CD, PIK3CB
56	hsa04912	GnRH signaling pathway	5	87	1.02	0.00047	MAPK1, EGFR, MAP2K1, GNAS, MAPK9
57	hsa04550	Signaling pathways regulating pluripotency of stem cells	6	141	0.89	0.00048	MAPK1, PIK3CA, MAP2K1, GSK3B, PIK3CD, PIK3CB
58	hsa04022	cGMP-PKG signaling pathway	6	163	0.83	0.001	MAPK1, MAP2K1, EDNRA, ADORA1, PIK3CG, KNG1
59	hsa04730	Long-term depression	4	59	1.09	0.0012	MAPK1, MAP2K1, GNAS, BRAF
60	hsa04213	Longevity regulating pathway—multiple species	4	61	1.08	0.0013	PIK3CA, MTOR, PIK3CD, PIK3CB
61	hsa05120	Epithelial cell signaling in Helicobacter pylori infection	4	65	1.05	0.0016	EGFR, CXCR1, CXCR2, MAPK9
62	hsa05130	Pathogenic Escherichia coli infection	6	187	0.77	0.0019	MAPK1, TAB1, CASP8, MAP3K7, ABL1, MAPK9
63	hsa04728	Dopaminergic synapse	5	126	0.86	0.0022	SLC6A3, GSK3B, GNAS, GNB1, MAPK9
64	hsa00562	Inositol phosphate metabolism	4	72	1.01	0.0023	PIK3CA, PIK3CD, PIK3CG, PIK3CB
65	hsa04115	p53 signaling pathway	4	72	1.01	0.0023	CDK2, CASP8, CDK1, CHEK1
66	hsa01100	Metabolic pathways	18	1435	0.36	0.0024	PYGL, ALOX12, CYP2C9, PIK3CA, ALOX15, AOC3, PNP, PDE10A, HSD11B1, CYP17A1, CYP2C19, ALOX5, PIK3CD, DHFR, FBP1, PIK3CG, CYP3A4, PIK3CB
67	hsa05418	Fluid shear stress and atherosclerosis	5	129	0.85	0.0024	PIK3CA, MAP3K7, PIK3CD, MAPK9, PIK3CB
68	hsa04371	Apelin signaling pathway	5	133	0.84	0.0026	MAPK1, MAP2K1, MTOR, GNB1, PIK3CG
69	hsa05017	Spinocerebellar ataxia	5	135	0.83	0.0028	PIK3CA, MTOR, PIK3CD, MAPK9, PIK3CB
70	hsa04217	Necroptosis	5	147	0.79	0.0039	PYGL, ALOX15, CASP8, PARP1, MAPK9
71	hsa04921	Oxytocin signaling pathway	5	147	0.79	0.0039	MAPK1, EGFR, MAP2K1, GNAS, PIK3CG
72	hsa04211	Longevity regulating pathway	4	87	0.92	0.0041	PIK3CA, MTOR, PIK3CD, PIK3CB
73	hsa04916	Melanogenesis	4	95	0.89	0.0055	MAPK1, MAP2K1, GSK3B, GNAS
74	hsa04064	NF-kappa B signaling pathway	4	101	0.86	0.0066	TAB1, PARP1, MAP3K7, SYK
75	hsa04621	NOD-like receptor signaling pathway	5	173	0.72	0.0073	MAPK1, TAB1, CASP8, MAP3K7, MAPK9
76	hsa00140	Steroid hormone biosynthesis	3	60	0.96	0.012	HSD11B1, CYP17A1, CYP3A4
77	hsa04114	Oocyte meiosis	4	121	0.78	0.012	MAPK1, CDK2, MAP2K1, CDK1
78	hsa04720	Long-term potentiation	3	63	0.94	0.0136	MAPK1, MAP2K1, BRAF
79	hsa00982	Drug metabolism—cytochrome P450	3	64	0.93	0.0141	CYP2C9, CYP2C19, CYP3A4
80	hsa00980	Metabolism of xenobiotics by cytochrome P450	3	69	0.9	0.0167	CYP2C9, HSD11B1, CYP3A4
81	hsa04622	RIG-I-like receptor signaling pathway	3	69	0.9	0.0167	CASP8, MAP3K7, MAPK9
82	hsa04721	Synaptic vesicle cycle	3	72	0.88	0.0181	SLC6A2, SLC6A4, SLC6A3
83	hsa04310	Wnt signaling pathway	4	154	0.68	0.0246	MMP7, GSK3B, MAP3K7, MAPK9
84	hsa04610	Complement and coagulation cascades	3	82	0.82	0.0251	BDKRB1, F8, KNG1
85	hsa04713	Circadian entrainment	3	91	0.78	0.032	MAPK1, GNAS, GNB1
86	hsa04070	Phosphatidylinositol signaling system	3	94	0.77	0.0346	PIK3CA, PIK3CD, PIK3CB

13 neurological pathways are found such as Neuroactive ligand–receptor interaction (hsa04080) Serotonergic synapse (hsa04726) cAMP signaling pathway (hsa04024) Morphine addiction (hsa05032) Neurotrophin signaling pathway (hsa04722) GABAergic synapse (hsa04727) Retrograde endocannabinoid signaling (hsa04723) Dopaminergic synapse (hsa04728) Axon guidance (hsa04360) Long-term depression (hsa04730) Long-term potentiation (hsa04720) Synaptic vesicle cycle (hsa04721) Circadian entrainment. We have utilized KEGG analysis for the respective gene annotations.

## Discussion

The pharmacokinetic, toxicological, bioactivity score, docking, and molecular dynamic assessments presented in this research article collectively contribute to a comprehensive understanding of Asarinin's potential as a therapeutic agent, particularly in the context of migraine management. The findings provide valuable insights into Asarinin's pharmacological properties, safety profile, and potential interactions with the CGRP. The pharmacokinetic profile of Asarinin reveals that its clearance, enzyme interactions, and predicted safe daily intake contribute to its overall safety profile. The negative clearance value of − 0.126 ml/min/kg signifies that the elimination process is overshadowed by the absorption process. It indicates a scenario where the rate of drug absorption exceeds its elimination rate during the specified experimental conditions^49^. The high gastrointestinal absorption, P-glycoprotein I inhibition^50,51^, and enhanced central nervous system permeability suggest promising pharmacokinetic characteristics for Asarinin's effectiveness in addressing migraine. The absence of mutagenic, carcinogenic, irritant, or reproductive effects, as well as a clean bill of health from the OSIRIS property explorer^52^, establishes Asarinin as a potentially safe therapeutic option. Quantitative Estimate of Druglikeness values can range between 0 and 1 considered as safest compound^53^. The drug-likeness score of asarinin 0.65 further supports its compatibility with commonly available drugs, enhancing its potential for clinical development. The bioactivity score assessment, conducted through Molinspiration, adds another layer of evidence supporting Asarinin's therapeutic potential. It is having good bioactivity score across important drug targets, along with the absence of PAINS warnings, suggests that Asarinin possesses desirable medicinal chemistry features. This is a positive indicator for its effectiveness in addressing specific targets related to migraine pathology. The docking assessment, utilizing molecular docking simulations, provides a theoretical foundation for understanding how Asarinin interacts with the CGRP receptor. The formation of hydrogen bonds with specific amino acid residues such as LYS2103, ARG2119, THR2120, and THR2122 in CGRP, as evidenced by the docking results, suggests a potential mechanism for Asarinin's activity. The favourable binding energy strengthens the hypothesis that Asarinin (− 10.03 kcal/mol) could have a significant ability to interact with CGRP. The molecular dynamic assessment extends the understanding gained from docking studies by exploring the dynamic behavior of Asarinin-CGRP complexes over time. RMSD and interaction analysis shed light on the stability and nature of interactions between Asarinin and CGRP. Gibb’s free energy lower than − 25 kcal/mol is considered as good indicative of strong binding affinity. The results suggest that Asarinin (Δ*G* Bind =  − 39.4678 kcal/mol) is potentially a more effective binder to the target than Atogepant (Δ*G* Bind =  − 34.84066 kcal/mol) due to its lower overall binding free energy. This finding is particularly relevant for drug design, where binding affinity is a critical parameter. Atogepant's higher ligand strain energy indicates that it undergoes more structural deformation upon binding, which could affect its stability and efficacy. Additionally, its less favourable solvation energy suggests greater desolvation penalties, which can impact its binding affinity. On the other hand, Asarinin's lower strain energy and solvation penalties contribute significantly to its stronger binding affinity. Overall, this analysis highlights the importance of evaluating multiple energy contributions to understand the binding characteristics of potential drug candidates fully. Asarinin emerges as a promising candidate due to its favourable binding free energy profile, but there may be room for improvement in its molecular interactions to enhance its efficiency and overall therapeutic potential.

**Fig. 6:**
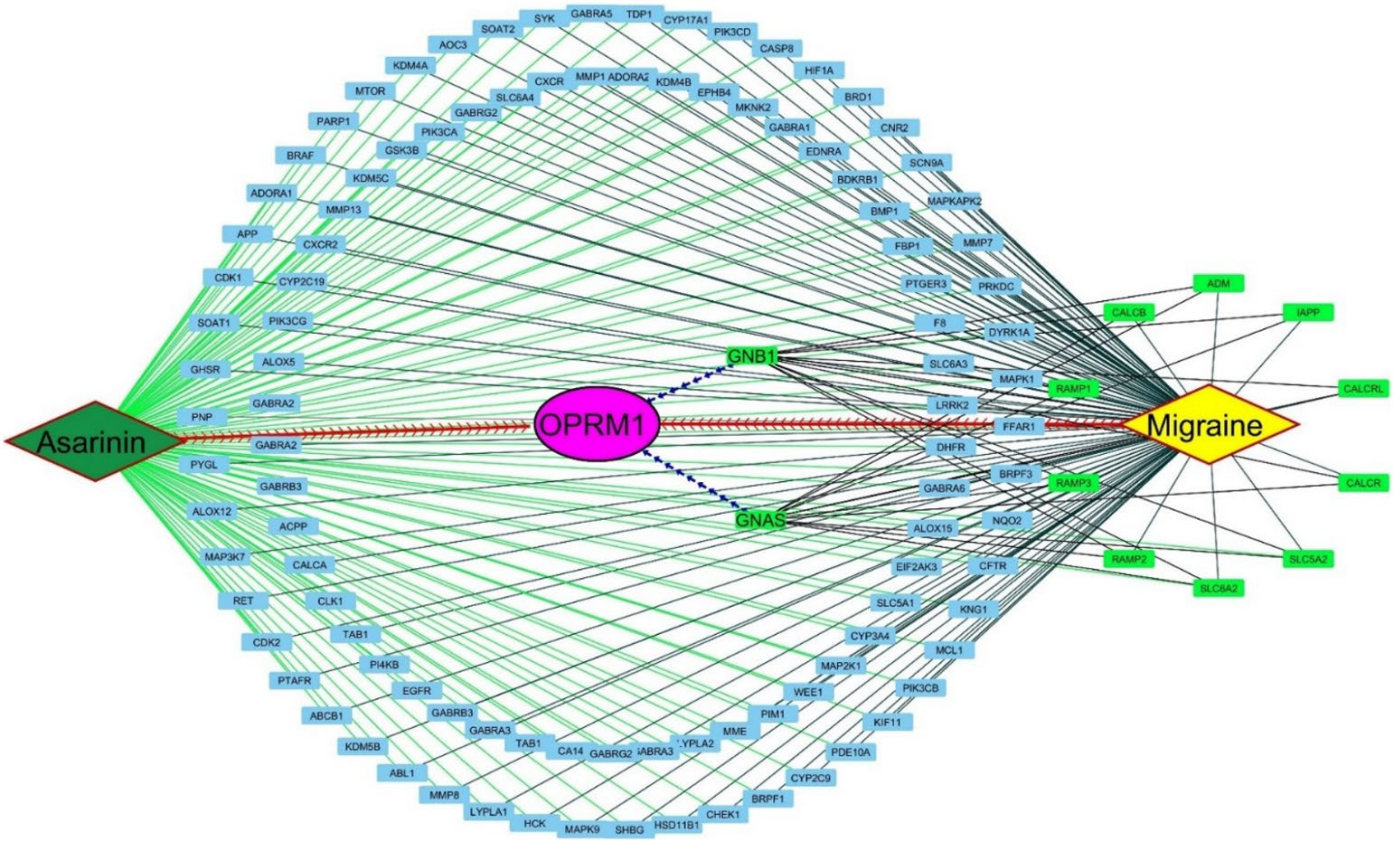
The figure illustrates a detailed protein interaction network related to migraines, with Asarinin highlighted as a novel biomolecule targeting multiple proteins. Asarinin (green diamond) is connected to a wide array of proteins (blue rectangles) via green edges, indicating its multifaceted influence. Central to this network is OPRM1 (purple oval), serving as a key intermediary linking Asarinin to migraine (yellow diamond) through direct red dashed arrows. Additional proteins like GNB1 and GNAS (green rectangles) further connect OPRM1 to migraine-related pathways. The involvement of proteins such as MTOR, PIK3CA, EGFR, ALOX5, ALOX12, and ALOX15 underscores the network’s complexity and highlights the roles of cell growth, metabolism, and inflammatory responses in migraine pathophysiology. This comprehensive network demonstrates Asarinin’s potential as a multifunctional therapeutic agent, providing valuable insights into the molecular mechanisms of migraines and identifying new avenues for treatment. We utilized CytoScape_v3.10.0 software to create this figure.

The network analysis suggested that the specific proteins such as OPRM1, GNB1, and GNAS are interlinked and interacted with the migraine with aura pathological targets like RAMP1^54^, RAMP2^55^, RAMP3^56^, CALCR^57^, CALCB^58^, ADM^59^, IAPP^60^, and SLC5A2^61^. OPRM1 (Opioid Receptor Mu 1) opioid receptors are involved in pain modulation^62^, and there is evidence suggesting that the endogenous opioid system may play a role in migraine pathophysiology. GNB1 (G Protein Subunit Beta 1) G proteins are signaling molecules involved in transmitting signals from the cell surface to the inside of the cell. Abnormalities in signaling pathways may contribute to migraine susceptibility^63^. GNAS (G Protein Subunit Alpha S) similar to GNB1 is involved in G protein signaling. Genetic variations in GNAS have been associated with certain migraine subtypes^64^. RAMP1, RAMP2, RAMP3 (Receptor Activity Modifying Proteins) proteins are involved in the modulation of receptors, such as the calcitonin receptor-like receptor (CALCR), which plays a role in vascular regulation. Abnormalities in vascular function are thought to contribute to migraine attacks. CALCR (Calcitonin Receptor) is associated with the calcitonin gene-related peptide (CGRP) receptor, which is implicated in migraine^65^. CGRP is a neuropeptide involved in vasodilation and neurogenic inflammation, both of which are thought to play a role in migraine attacks^66^. CALCB (Calcitonin-Related Polypeptide Beta) is another component related to CGRP, and variations in the CGRP pathway are being explored as potential targets for migraine treatment^67^. SLC5A2 (Sodium/glucose cotransporter 2) is involved in glucose and sodium transport. While there is not a direct known link to migraines, disturbances in ion transport can impact neuronal excitability and may have implications in migraine pathophysiology^68^. The analysis of protein interactions indicates a network where these proteins are interconnected. This network analysis revealed that OPRM1 serves as a central hub, initiating the activation of GNAS and GNB1 which is illustrated in Fig. 6. Eight out of thirteen recognized brain circuits are significant triggers in migraines. Among these are the GABAergic synapse, the Dopaminergic synapse, the Serotonergic synapse, the cAMP signaling pathway, the Neurotrophin signaling pathway, the Retrograde endocannabinoid signaling, and the Circadian entrainment pathways. In the onset and maintenance of migraine headaches, these pathways emphasize the intricate interaction of neurotransmitters, receptors, and signaling pathways. This activation, in turn, influences other proteins like RAMP 1–3, CALCB, CALCR, and SLC5A2, which are implicated in migraine attacks. By directing Asarinin toward OPRM1, it has the potential to inhibit OPRM1, further reducing the synthesis of CGRP and the activation of pain stimulation from other biomarkers implicated in migraine attacks. This suggests that Asarinin could play a pivotal role as a potential future for individuals suffering from migraines, offering the prospect of alleviating symptoms without causing severe adverse effects. It's important to note that while natural products may offer relief for some individuals, their efficacy varies, and not all remedies work for everyone. Additionally, maintaining a healthy lifestyle, managing stress, and identifying and avoiding triggers are essential components of a holistic approach to migraine management.

## Future implications

Looking ahead, the multifaceted assessments conducted in this research article pave the way for a promising future in harnessing Asarinin as a therapeutic agent, particularly in the realm of migraine management. The integration of pharmacokinetic, toxicological, bioactivity score, docking, and molecular dynamic analyses provides a holistic perspective that not only elucidates Asarinin's pharmacological properties but also underscores its potential as a novel and effective intervention for migraines. The amalgamation of diverse assessments sets the stage for future research, preclinical investigations, and potential clinical trials that would be instrumental in establishing the safety and efficacy of Asarinin in human subjects, marking Asarinin as a beacon of hope in the pursuit of more effective migraine therapies. A thorough toxicological assessment is crucial for determining the safety of Asarinin before clinical trials.

## Conclusion

In conclusion, the comprehensive array of assessments conducted in this research affords a thorough understanding of Asarinin's potential as a therapeutic agent in the context of migraine management. The combined findings contribute valuable insights into its pharmacological properties, safety profile, and potential interactions with the CGRP receptor. Its favourable pharmacokinetic profile, safety in toxicological evaluations, and potent bioactivity against CGRP suggest effectiveness. These endeavours will be instrumental in establishing the safety and efficacy of Asarinin in human subjects, marking it as a beacon of hope in the pursuit of more effective migraine therapies. Asarinin emerges as a promising candidate, offering a multifaceted approach to addressing the complex landscape of migraine management. OPRM1 emerges as a pivotal hub, instigating the activation of GNAS and GNB1, subsequently influencing proteins such as RAMP1, RAMP2, RAMP3, CALCB, CALCR, and SLC5A2 all implicated in migraine attacks. Significantly, OPRM1 stands out as a crucial target in network analysis presenting a promising avenue for intervention. Asarinin holds promise as a therapeutic agent, offering a multifaceted approach to addressing the intricate landscape of migraine management by targeting OPRM1. These findings pave the way for further exploration and potential clinical applications, marking Asarinin as a beacon of hope in the pursuit of more effective migraine therapies.

## Data Availability

All data generated or analysed during this study are included in this published article.
